# Eine Fallstudie – Zum Verhältnis von Medikation und geheimpolizeilicher Praxis im MfS-Haftkrankenhaus Berlin-Hohenschönhausen

**DOI:** 10.1007/s00115-025-01849-z

**Published:** 2025-06-13

**Authors:** Tillmann Wickert, Kathleen Haack, Felicitas Söhner, Ekkehardt Kumbier

**Affiliations:** Arbeitsbereich Geschichte der Medizin, Universitätsmedizin Rostock, Doberaner Straße 140, 18057 Rostock, Deutschland

## Hintergrund

Haben Ärzte des MfS^1^-Haftkrankenhauses Hohenschönhausen die Gabe von Medikamenten veranlasst, die das Aussageverhalten und die Vernehmungsfähigkeit von Häftlingen beeinflusste? Entsprechende Annahmen ehemaliger Häftlinge konnten bislang weder juristisch noch wissenschaftlich erhärtet werden [3]. Doch neue Einblicke in Ermittlungs‑, Haft- und Krankenakten im Bundesarchiv (BStU) von Häftlingen des MfS-Krankenhauses Hohenschönhausen bestätigen eine solche Annahme. So enthalten etwa die „Stasiakten“ des Häftlings Clausmann^2^ erstmals schriftliche Hinweise auf die gezielte Verschleierung der Medikation durch das MfS. Es kann davon ausgegangen werden, dass eine solche als geheimpolizeiliches Instrument angesehen und entsprechend gehandhabt wurde.

## Ein „Hobby-Anwalt“ im Visier des MfS

Im April 1986 wurde der 37-jährige Clausmann verhaftet und in die MfS-Untersuchungshaftanstalt in Halle gebracht. Vorgeworfen wurde ihm das Sammeln und Verbreiten von „Nachrichten, die geeignet sind, den Interessen der DDR zu schaden“ [10Bl. 255] – Vorwürfe, die das MfS zur strafrechtlichen Verfolgung von Ausreiseantragstellern verwendete [2, S. 313]. Clausmann selbst hatte keinen Ausreiseantrag gestellt, aber andere beraten [5Bl. 86–89]. Das war prinzipiell nicht illegal, was auch die Stasi wusste. Sie musste fürchten, nichts Relevantes gegen Clausmann in der Hand zu haben. Den fehlenden Baustein lieferte die Vernehmung eines wegen Ausreisebemühungen inhaftierten Ehepaars. Dieses sagte gegenüber dem MfS aus, Clausmann habe ihm bei der Formulierung und Übermittlung eines Briefs an den bayerischen CSU-Politiker Franz Josef Strauß geholfen. Damit sah das MfS einen Anfangsverdacht auf das „Verbreiten von Nachrichten, die geeignet sind, die Interessen der DDR im Ausland zu schädigen“ (§ 219 StGB) und leitete Ermittlungen gegen Clausmann ein [5Bl. 103]. Doch die Kriminalisierungsstrategie des MfS stand auf tönernen Füßen. Benötigt wurde ein Geständnis von Clausmann [9, 102–105]. Dieser widersetzte sich und beteuerte seine Unschuld [5Bl. 14]. Daraufhin erweiterte das MfS die Anklage um § 220, Abs. 1 „Öffentliche Herabwürdigung staatlicher Organe“ [7Bl. 54]. Grundlage war ein Disput mit der Staatsanwältin. Clausmann unterstellte dieser Befangenheit und unterstrich die Gesetzwidrigkeit seiner Inhaftierung. Explizit zum Vorwurf des § 220 wurde eine Erweiterungsvernehmung angesetzt, die im Haftkrankenhaus der MfS-Untersuchungshaftanstalt Berlin-Hohenschönhausen erfolgte, wohin Clausmann wegen eines Schulter-Arm-Syndroms nach einer Bandscheibenoperation verlegt worden war.

## Im Haftkrankenhaus

Im Haftkrankenhaus blieb er neun Wochen und wurde fünfmal vernommen. In den drei Tagen vor der besagten Erweiterungsvernehmung am 12.06. wurde Clausmann auffällig stark medikamentiert (Tab. 1). Neben Schmerzmitteln erhielt er verschiedene sedierende Medikamente wie u. a. Benzodiazepine (Medazepam, Nitrazepam, Diazepam) und andere Tranquilizer sowie ein Antihistaminikum, die in Interaktion zu Müdigkeit und Konzentrationsstörungen führen [4Bl. 27].

**Tab. 1:** Eintrag der Medikamentengabe und Nebenwirkungen im Krankenblatt des Haftkrankenhauses der MfS-Untersuchungshaftanstalt Berlin-Hohenschönhausen

Dienstag, 10. Juni	Mittwoch, 11. Juni	Donnerstag, 12. Juni
3 × 1 Rudotel® 1-1‑2 Rewodina® 1 × 1 Indometacin® (21 Uhr) Bei Bedarf in der Nacht bis 3 × 1 Radedorm®	3 × 1 Rudotel® 1-1‑2 Rewodina® 1 × 1 Indometacin® (21 Uhr) 1 Amp. Faustan® (Injektion) 3 × 1 Metrobamat® 20 Tropfen Valorcordin® *Eintrag im Krankenblatt: Nach Medizingabe erbrochen, Herzbeschwerden, Kreislaufbeschwerden*	3 × 1 Metrobamat® Metrobamat® (20 Uhr) Vernehmung (Beginn: 8:15 Uhr; [6Bl. 137])

Über die Dosierung sowie die Indikation wird in den Krankenunterlagen nichts angegeben. Allerdings traten bei Clausmann erhebliche Nebenwirkungen mit Kreislaufproblemen und Erbrechen auf [4Bl. 27]. Auch ein handschriftlicher Vermerk des Untersuchungsführers vom Vernehmungstag verweist auf nicht näher beschriebene „Reaktionen“ Clausmanns, weswegen der behandelnde Psychiater des Haftkrankenhauses hinzugezogen wurde. Dieser erwähnte die Medikation des Vortages nicht und hielt auch eine forensisch-psychiatrische Begutachtung für nicht notwendig [9Bl. 171]. Clausmann selbst äußerte drei Wochen später, seine Vernehmungsfähigkeit in der Vernehmung am 12.06. sei durch die Medikation beeinträchtigt gewesen. Er hätte die Kontrolle über sein Aussageverhalten verloren. An seine Angaben im verschriftlichten Protokoll könnte er sich nicht erinnern [4Bl. 17]. Er forderte, den behandelnden Nervenarzt des Haftkrankenhauses zu sprechen. Zur Vernehmung hinzugezogen erklärte dieser, dass es „sich bei den verabreichten Medikamenten um schmerzstillende und beruhigende Tabletten handelte, die keinen Einfluss auf seine geistige Aktivität […] hatten“ [8Bl. 53]. Grundsätzlich wären die Medikamente „bestenfalls geeignet, eine raschere Ermüdbarkeit zu erzeugen“ [8Bl. 58]. Von der Einschätzung des Psychiaters unbeirrt, hielt Clausmann an seinen Vorwürfen fest und forderte die Tonbandaufzeichnung der Vernehmung vom 12.06. anzuhören [8Bl. 57]. Eine Wiedergabe des Mitschnitts war angeblich „aus technischen Gründen“ nicht möglich [9Bl. 9]. Tage später versuchte Clausmann, seiner Frau Hinweise zukommen zu lassen. In einem Brief formulierte er: „Seit dem zweiten Sprecher nehme ich keine Medikamente mehr. Um welche Medikamente es sich bisher gehandelt hat, darf mir der Neurologe bzw. Psychiater nicht sagen“ [7Bl. 100]. Auch seinem Anwalt muss Clausmann die Gabe der Medikation mitgeteilt haben. Auf dessen Nachhaken sollte eine Auflistung aller während des Haftkrankenhausaufenthalts verabreichten Medikamente erfolgen. Daraufhin mahnte die Abt. IX der Bezirksverwaltung Halle die Haftkrankenhausleitung an, gegenüber dem Gericht keine Angaben über die Wirkungsweise der Medikamente zu machen [4Bl. 9]. Mitgeteilt wurde der Staatsanwaltschaft lediglich, dass die Behandlung „sowohl pharmakologisch als auch physiotherapeutisch kompliziert“ war [4Bl. 15].

## Diskussion

Der Fall Clausmann enthält erstmals Hinweise darauf, dass das MfS psychotrop wirkende Substanzen bei Untersuchungshäftlingen im Vorfeld der Vernehmung einsetzte und die Gabe gezielt verschleierte. Für die Schmerzbehandlung wurden Diclofenac (Rewodina®) und Indometacin gegeben. Psychotrop wirkten Medazepam (Rudotel®), Diazepam (Faustan®), Nitrazepam (Radedorm®) und Meprobomat. Das Verhör unter dieser Medikamentenkombination war sicherlich problematisch. Es muss angenommen werden, dass der Inhaftierte vor allem in kognitiver Hinsicht deutlich beeinträchtigt war. Er selbst fühlte sich in der Vernehmung „gedämpft“. Es handelt sich bei den Medikamenten um drei verschiedene Benzodiazepine sowie mit Valocordin® um Phenobarbital und Bromisoval und das ebenfalls sedierende Meprobomat [1]. Die Dosierungen der Präparate lagen vermutlich im mittleren Bereich. Das Problem war die kombinierte Gabe in kurzer Zeit. Das ist vor allem hinsichtlich der Halbwertzeiten mit möglichen Interaktionen ein Problem. Somit kann von einer Potenzierung der sedierenden Wirkung ausgegangen werden. Die verzeichneten „Herz- und Kreislaufbeschwerden“ können als Nebenwirkungen im Sinne einer orthostatischen Dysregulation eingeordnet werden und das Erbrechen dürfte durch Diclofenac und vor allem Indometacin verursacht worden sein.

Der Umgang mit der Medikation in dieser Situation legt nahe, dass das MfS diese als ermittlungstaktisches Instrument verstand. Denn die Erweiterungsvernehmung war für die Gewinnung eines Geständnisses und damit die weitere Anklage äußerst wichtig. Die Rolle des MfS-Psychiaters im Haftkrankenhaus und die mögliche Bereitschaft, seine Expertise im Rahmen dieser repressiven Praxis einzusetzen, ist ein zentraler Punkt für die weitere psychiatriehistorische Forschung.

## Fazit für die Praxis

Es bleibt zu prüfen, inwieweit diese medizinische Einflussnahme ein Teil einer breiteren, systematischen Strategie des MfS war. Die Forschung sollte sich insbesondere auf die noch kaum erforschten Bereiche des Zentralen Medizinischen Dienstes und dessen Verknüpfungen zu den operativen Strukturen des MfS konzentrieren, um diese Aspekte der DDR-Repressionsmethoden umfassend zu verstehen.

## Footnotes

1 MfS, Abkürzung für Ministerium für Staatssicherheit der DDR. Im Folgenden wird auch die Kurzform Stasi verwendet.

2 Der Name Clausmann ist fiktiv und dient im Folgenden als Pseudonym für den Namen C.-D. G.
